# Conservative Management of an Iatrogenic Esophageal Tear in Kenya

**DOI:** 10.1155/2015/102540

**Published:** 2015-07-14

**Authors:** Peter Waweru, David Mwaniki

**Affiliations:** ^1^Department of Surgery, St. Mary's Mission Hospital, P.O. Box 3409, Nairobi 00506, Kenya; ^2^Department of Surgery, The Karen Hospital, P.O. Box 74240, Nairobi 00200, Kenya

## Abstract

Since its description over 250 years ago, diagnosis of esophageal perforation remains challenging, its management controversial, and its mortality high. This rare, devastating, mostly iatrogenic, condition can quickly lead to severe complications and death due to an overwhelming inflammatory response to gastric contents in the mediastinum. Diagnosis is made with the help of esophagograms and although such tears have traditionally been managed via aggressive surgical approach, recent reports emphasize a shift in favor of nonoperative care which unfortunately remains controversial. We here present a case of an iatrogenic esophageal tear resulting from a routine esophagoscopy in a 50-year-old lady presenting with dysphagia. The esophageal tear, almost missed, was eventually successfully managed conservatively, thanks to a relatively early diagnosis.

## 1. Introduction

Esophageal perforation is a rare, devastating, and often life-threatening clinical condition [1] typically resulting from endoscopic procedures [2]. This condition remains difficult to diagnose and manage and can quickly cause death without alarm [3], owing to its nonspecific and varied clinical symptomatology [1]. While surgery has been the mainstay of treatment, nonoperative management approaches for this condition are becoming more and more common [4], but they remain controversial.

We present a case of an iatrogenic esophageal perforation that developed after a diagnostic esophagoscopy in a female patient with odynophagia and the subsequent conservative treatment after an almost missed diagnosis. In view of the recent but controversial emphasis on nonoperative treatment, this case has been presented to add to the repertoire of success stories, thus encouraging nonoperative care, even in developing countries.

## 2. Case Report

A 50-year-old lady presented with dysphagia, odynophagia, and regurgitation of foods. Although an esophagogastroduodenoscopy (OGD) done previously had shown gastroesophageal reflux disease (GERD), resolving esophagitis and gastritis, this new onset dysphagia warranted further examination. A barium swallow, postnasal space and chest computed tomography (CT) scans were all normal. An indirect laryngoscopy was attempted but unsuccessful due to a strong gag reflex and consequently a direct laryngoscopy and esophagoscopy were done. The investigations revealed laryngeal erythema and gastric fundal erosion with no other abnormalities. After esophagoscopy, she was successfully reversed, observed in the postanesthetic care unit, and eventually discharged to the ward in stable conditions.

**Figure 1:**
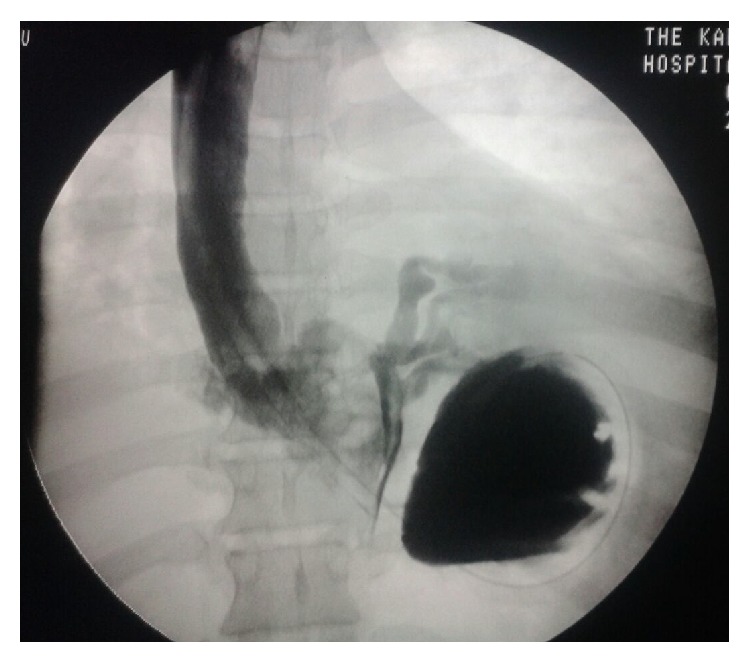
Gastrografin swallow showing leak of contrast into the left mediastinum and left pleural cavity.

In the ward, she suddenly developed severe epigastric pains, respiratory distress, and difficulty in speaking, for which she was given intravenous (IV) Esomeprazole 80 mg and Buscopan (hyoscine butylbromide) 40 mg for what appeared like acute exacerbation of gastritis. She was also started on oxygen. There being minimal improvement, she was immediately transferred to the intensive care unit, where close monitoring and oxygen therapy were continued. Further investigations included an electrocardiogram (ECG) and echocardiogram which were both normal and a CT scan of the chest which revealed severe basal pneumonia. A gastrografin swallow was finally done (Figure 1) and showed leakage of the contrast into the mediastinum and left pleural cavity.

Following the diagnosis of an esophageal perforation, a decision was made to manage the patient nonoperatively considering the relatively early diagnosis (few hours after esophagoscopy). A chest drain was inserted percutaneously and a nasogastric tube (NGT) inserted to rest the esophagus and drain the gastric contents. She was kept* nil per oral (NPO)* and was started on broad-spectrum IV antibiotics, oxygen, IV proton pump inhibitors, IV fluids, and analgesics.

**Figure 2:**
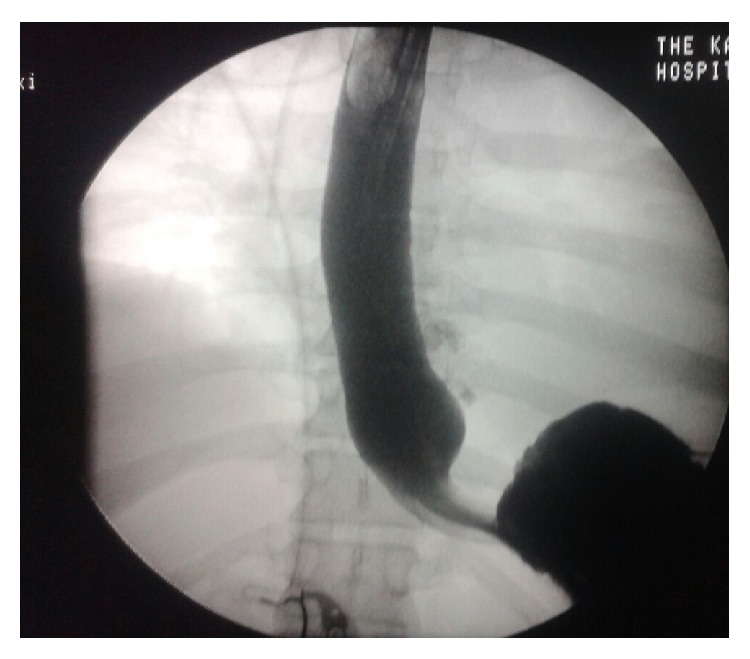
Follow-up gastrografin swallow showing reduced leakage.

A follow-up gastrografin swallow done on day 12 after esophagoscopy showed notable reduced leakage (Figure 2).

Later, a repeat OGD was carefully performed on day 14 to review the status of the injury and showed a 2 cm tear at 30 cm in the posterior wall that was contracting. The patient showed good progress on conservative management and was transferred to the ward on day 15. Feeding was gradually advanced from total parenteral to feeding via NGT to oral sips and finally solid meals before she was discharged home after about one month in stable conditions.

## 3. Discussion

Esophageal perforation, reported as early as the 18th century (Hermann Boerhaave, 1724) [5], is a rare and often grave clinical condition [4] with high mortality rates over 40%, especially in septic patients [6]. While the true incidence is unclear [4], the majority of esophageal rupture cases (up to 59%) are iatrogenic [1] resulting from esophagoscopy [2] despite the actual risk of esophageal perforation during endoscopy being low [2, 7]. Boerhaave syndrome, a spontaneous esophageal rupture with no preexisting pathology, accounts for about 15% of the cases [8]. Foreign-body ingestion accounts for 12% of the cases, trauma 9%, operative injury 2%, tumors 1%, and other causes 2% [8].

Thoracic esophageal perforations occur frequently [1, 8] and can lead to serious complications and death without alarm [3, 9], owing to the mediastinal contamination that ensues soon after the perforation [7]. This contamination, which is exacerbated by the negative intrathoracic pressure that draws esophageal contents into the mediastinum [10], evokes an overwhelming inflammatory response [11] leading to mediastinitis, initially chemical mediastinitis, followed by bacterial invasion and severe mediastinal necrosis [7]. Eventually, sepsis ensues leading to multiple-organ failure and death [3, 4]. The extent of this inflammation (mediastinitis), and thus the morbidity and mortality of esophageal perforation, depends not only on the cause and location of the perforation but also on the time interval between onset and access to appropriate treatment [3, 12]. It has been shown that early detection reduces mortality by over 50% [11] and treatment delays over 24 hours increase mortality significantly [13]. Unfortunately, prompt diagnosis continues to be exigent for most clinicians [5].

Diagnosis of esophageal perforation is challenging owing to a nonspecific and varied clinical presentation [1] that mimics a myriad of other disorders such as myocardial infarction and peptic ulcer perforation [14]. Patients may present with any combination of nonspecific signs and symptoms including fever, tachycardia, tachypnea, acute onset chest pain, dysphagia, vomiting, and shortness of breath [4, 6, 15]. A high index of suspicion is therefore needed for recognition of esophageal perforation [5]. Once suspected, patients should be evaluated quickly with a combination of radiographs and esophagograms [8, 14]. Accurate diagnosis may however require added investigations including computed tomography and flexible esophagoscopy [7, 12].

Treatment of esophageal perforations remains a challenge [13] and the appropriate management is controversial [9]. Traditionally, surgery has been the mainstay of treatment [14], but recent reports emphasize a shift in treatment strategies with nonoperative approaches becoming more common [4, 9]. It has been shown that, with careful patient selection, nonoperative management can be the treatment of choice for esophageal perforations [6] with good outcomes [9, 12, 15, 16]. Altorjay et al. [17] and others have suggested criteria for selection of nonoperative treatment including early perforations (or contained leak if diagnosis delayed); leak draining back to the esophagus; nonseptic patients; perforation not involving a neoplasm, abdominal esophagus, or distal obstruction; and availability of an experienced thoracic surgeon and contrast. When these established guidelines are followed, survival rates of up to 100% have been reported [7, 9, 15].

Patients selected for nonoperative treatment are started on broad-spectrum antibiotics, intravenous fluids, oxygen therapy, adequate analgesia, and gastric acid suppression and kept nil by mouth in an intensive care unit [4, 18]. A nasogastric tube is placed to clear gastric contents and limit further contamination [9] and mediastinal contamination drained percutaneously/radiologically [18] via the chest tubes, thereby converting the esophageal perforations to esophagocutaneous fistulae that heal similar to gastrointestinal fistulae [6]. Apart from observation, the range of conservative management is growing, with the increasing use of endoscopic stents, clips, vacuum sponge therapy, and fibrin glue application [8, 12] for the selected patients. Notably though, even with meticulous patient selection, up to 20% develop multiple complications within 24 hours and require surgical intervention [2, 7].

In our patient, the diagnosis of an iatrogenic esophageal perforation was made relatively early and a multidisciplinary team chose conservative treatment as the treatment of choice given that the patient was not septic and had no contraindications to the treatment. This was instituted without complications, achieving good results. While there are few such reports in resource-limited settings, conservative management should be considered in the few hospitals with institutional capacities.
